# Clinical profile of Korean children with spina bifida: a single-center prospective cohort study

**DOI:** 10.1186/s12887-024-05229-5

**Published:** 2024-12-03

**Authors:** Hyeseon Yun, Seung Hyeon Yang, Hooyun Lee, Sang Woon Kim, Yong Seung Lee, Yoonhye Ji, Jieun Park, Jeong-Eun Ji, Eun Kyoung Choi

**Affiliations:** 1 College of Nursing and Brain Korea 21 FOUR Project, Yonsei University, Seoul, South Korea; 2 Department of Urology, Yonsei University College of Medicine, Seoul, South Korea; 3 Department of Nursing, Yonsei University Graduate School, Seoul, South Korea; 4 Department of Pediatric Urology, Bladder-Urethra Rehabilitation Clinic, Severance Children’s Hospital, Yonsei University Health System, Seoul, South Korea; 5 Graduate School of Nursing, Yonsei University, Seoul, South Korea; 6 College of Nursing and Mo-Im Kim Nursing Research Institute, Yonsei University, Seoul, South Korea

**Keywords:** Children, Cohort study, Prospective study, Spina bifida, Life Course Model

## Abstract

**Background:**

Spina bifida (SB) is a chronic condition requiring lifelong self-management, underscoring the need to establish a dedicated cohort for longitudinal monitoring of health outcomes. The purpose of this study was to describe the development and initial implementation of a single-center prospective cohort study of children with SB and their parents living in South Korea and to describe demographics, clinical outcomes, psychosocial characteristics, and family data for this cohort.

**Methods:**

This cohort was established through expert panel formation, identification of health indicators based on the Life Course Model for Spina Bifida, creation of a cohort database system, and quality control planning. Participants, children aged 4–12 years with SB and their parents, were recruited from a large SB clinic at a tertiary hospital in South Korea. Two approaches were used to collect data: (1) diagnosis and clinical outcomes (e.g., lesion level, surgical history, laboratory results) were collected from the electronic medical record by pediatric nurse practitioners and (2) demographics, psychosocial characteristics and family data were collected from online self-reported questionnaires completed by children with SB and their parents if the child with SB was aged 7–12 years and by only the parents if the child with SB was aged 4–6 years.

**Results:**

Between September 2022 and September 2023, 162 children (mean age 7.6 ± 2.6 years) and their parents participated, with 35.8% (4–6 years), 29% (7–9 years), and 35.2% (10–12 years). Diagnoses included lipomyelomeningocele (51.2%), myelomeningocele (27.2%), and tethered cord syndrome (20.4%). Clean intermittent catheterization was used by 38.3% and enemas by 22.2%. Moreover, 30.9% experienced urinary incontinence and 26.5% experienced fecal incontinence. Ambulation assistive devices were used by 14.8%, and ventriculoperitoneal shunts by only 4.3%.

**Conclusions:**

This cohort provides a comprehensive understanding of demographics, and clinical and psychosocial outcomes for children with SB in South Korea. The dataset offers opportunities for data-driven, life-course tailored interventions to meet the specific needs of this population and their families.

**Supplementary Information:**

The online version contains supplementary material available at 10.1186/s12887-024-05229-5.

## Introduction

Spina bifida (SB) is a neural tube defect and the most common central nervous system malformation [1]. Occurring early in fetal development, it leads to diverse central nervous system impairments, causing neurogenic bladder, neurogenic bowel, impaired sensation, limited mobility, orthopedic deformities, and variations in cognitive processing [1, 2]. Despite these functional challenges, advances in surgical techniques and effective bladder and bowel management methods have enabled most children with SB to survive into adulthood [3, 4]. The healthcare objectives for individuals with SB are to maintain optimal function throughout life, prevent secondary complications, and improve health-related quality of life (HRQoL) [5–7]. To improve the lifelong conditions and HRQoL of individuals with SB, acquiring self-management knowledge and skills, along with healthcare transitions across developmental stages, is crucial [5, 8, 9]. Longitudinal follow-up of health outcomes is necessary for a comprehensive understanding [5].

Based on the importance of longitudinal health outcome monitoring from a life course perspective [10], nationwide SB registries or prospective cohorts have been established in the United States, Canada, and Europe since the 2000s [11–14]. In the United States, the National Spina Bifida Patient Registry (NSBPR) commenced in 2008 encompassing 11,812 participants from 21 SB clinics. The registry collects data on demographics (age, race/ethnicity, insurance type), interventions (surgery, non-surgical procedures), and outcomes (bladder or bowel incontinence, mobility, skin problems) [15, 16]. Over the past 15 years, NSBPR has published numerous studies on the association between treatment and health outcomes based on data from the registry [17]. However, there are no SB cohorts or registries in Korea, lacking evidence regarding the long-term outcomes in Koreans with SB.

To date, previous studies [18–24] on SB published in Korea have shown a unique clinical distribution that differs from those of individuals with SB from other countries including the United States, Canada, and Sweden [11, 13–15, 26]. For example, in Korean studies, the ratio of myelomeningocele (MMC) to non-myelomeningocele (non-MMC) is about 4:6 or 3:7 [18, 21–24], whereas in the other countries mentioned above, the ratio is reversed [13–15, 26]. In addition, the proportion of people with SB who use wheelchairs or have hydrocephalus is only 5–10% in Korea [18, 21–24], while it is over 50% in other countries [13–15, 26]. However, regarding bladder and bowel management, the percentage of Korean children with SB who employed clean intermittent catheterization (CIC, 43.8%), used enemas (20.2%), and experienced urinary incontinence (52.0%) and fecal incontinence (36.0%) [18] are comparable to those in other countries [15, 27]. The unique clinical distribution of children with SB is remarkable, and establishing a cohort to track their long-term health outcomes would provide meaningful evidence.

In South Korea, a person’s disability is often perceived as an inferiority rather than an individual characteristic. This way of perceiving a person’s disability aligns with an East Asian interdependent viewpoint in which a person defines oneself in relation to others [19, 20]. Korean children with SB face challenges in effective self-management, particularly performing CIC at school, and struggle to conceal bladder or bowel dysfunction [19–21]. Ineffective self-management can lead to complications, such as kidney damage and increased frequency of urinary and fecal incontinence, which are associated with a lower HRQoL and delayed transition to adulthood [7, 19]. Clinically mild SB may render children more psychosocially vulnerable, emphasizing the importance of measuring psychosocial outcomes alongside clinical ones [5, 19]. Hence, longitudinal study of psychosocial indicators as well as the natural history of Korean children with SB is important to help understand their health problems.

In accordance with the Life Course Model for Spina Bifida [9], a combination of demographic, clinical, psychosocial, and family factors influence HRQoL in individuals with SB. Furthermore, Kinsman et al. (2000) highlighted the importance of establishing anticipatory guidance and transition planning across the lifespan based on comprehensiveness, coordination, and longitudinality [10]. Existing studies of Korean children with SB are cross-sectional studies. There is currently a need for longitudinal studies that are well-coordinated and comprehensiveness.

This single-center prospective cohort study of Korean children with SB was designed as a longitudinal endeavor, integrating clinical data from electronic medical records (EMRs) with demographic, psychosocial, and family data from an online self-report survey. This study aimed to describe the process of establishing and initially implementing a cohort of children with SB based on the Life Course Model of Spina Bifida [9]. The results of this study will provide a basis for the development of life-course tailored, person- and family-centered interventions to improve the HRQoL of children with SB and their families. The main objectives of this study were to describe the development and initial implementation of a single-center prospective study of children with SB and their parents living in South Korea and to describe demographics and clinical outcomes for children with SB in this cohort.

## Methods

### Study design

This is a single-center, prospective cohort study. This paper detailed the development, implementation, initial recruitment, and data collection of a cohort of children with SB and their parents in Korea and described the general and clinical characteristics of the children with SB in the cohort.

### Cohort development

#### Purpose of cohort development

This prospective cohort is the first cohort of children with SB and their parents in South Korea and was developed to provide an integrated database of demographics, clinical outcomes, and psychosocial characteristics, and family data for children with SB and their parents.

#### Research team and expert panel for cohort development

The research team for cohort development consisted of a professor of pediatric nursing, two doctoral research assistants, an undergraduate research assistant from Yonsei University College of Nursing, two professors of pediatric urology, and three pediatric nurse practitioners (PNPs) from the SB clinic at Severance Children's Hospital. The expert panel for cohort development consisted of two professors of pediatric urology, one professor of pediatric orthopedics, six PNPs (pediatric urology, neurosurgery, orthopedics, and general surgery), an epidemiologist in public health, and a statistician.

#### Theoretical framework for cohort development and selection of outcome variables

We employed Swanson's (2010) Life Course Model of Spina Bifida as the theoretical framework for cohort development. This model serves as a research framework, aiding children with SB in achieving desired adult outcomes. The dynamic process outlined in the model, divided into preschool, school-age, adolescence, and young adulthood, emphasizes milestones at each stage of child development, aligning with the International Classification of Functioning, Disability and Health’s perspective on functioning and participation rather than a medical, deficit-oriented focus on disease [9]. This alignment encompasses three domains: self-management/health (self-care), personal/social relationships, and employment/income support (major life areas). The model operates on the principle that the activities and participation of individuals with disabilities are the desired outcomes to be monitored and measured. This determination results from the interaction between impairments and personal and environmental factors [9]. Thus, we incorporated demographics and clinical characteristics of children with SB as inputs, selected HRQoL as outputs, and introduced family factors as environmental factors.

**Fig. 1:**
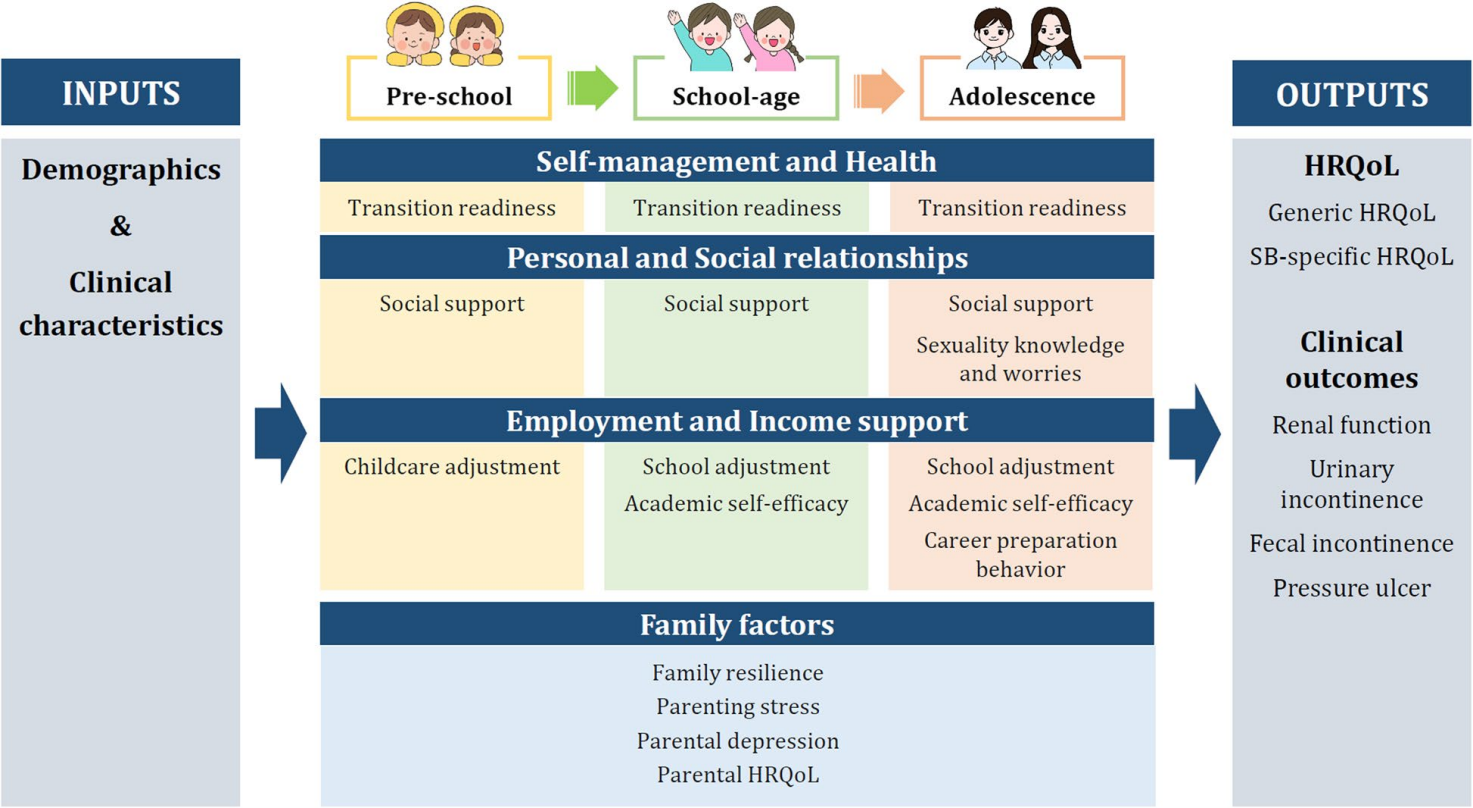
Theoretical framework for the cohort Note: HRQoL, health-related quality of life

We tentatively identified demographic, clinical, and psychosocial outcome variables impacting HRQoL in children with SB, drawing on variables used in prior publications from the NSBPR and other congenital and rare and incurable disease cohort studies. The research team iteratively selected these outcome variables through discussions and consultations with the expert panel. The detailed theoretical framework for this cohort and the outcome variables is depicted in Fig. 1.

#### Cohort database system

**Fig. 2:**
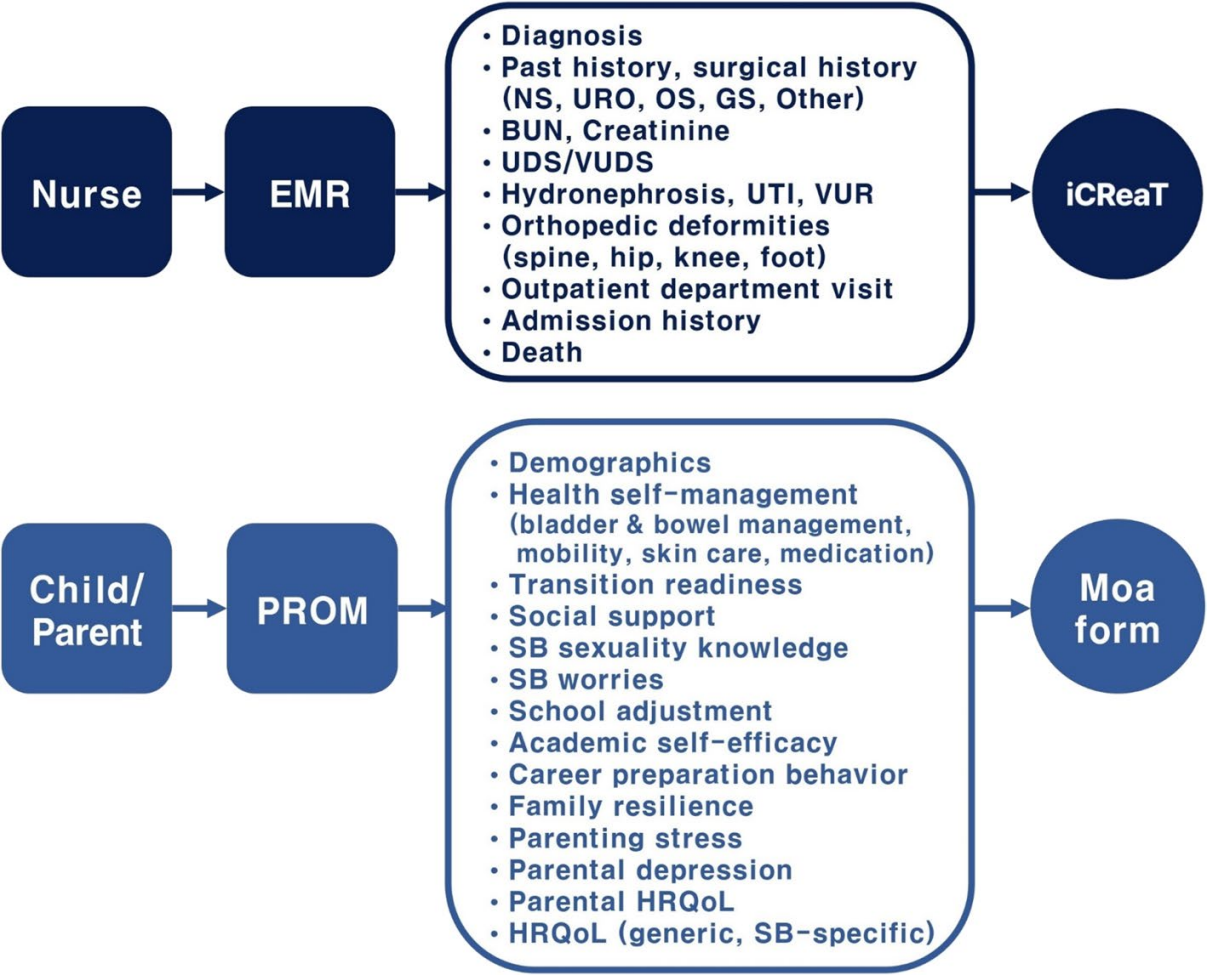
Cohort database system Note. BUN, blood urea nitrogen; EMR, electronic medical record; GS, general surgery; HRQoL, health-related quality of life; iCReaT, internet based clinical research and trial management system; NS, neurosurgery; OS, orthopedics; PROM, patient-reported outcome measure; SB, spina bifida; UDS, urodynamic study; UTI, Urinary tract infection; URO, urology; VUDS, video urodynamic study; VUR, vesicoureteral reflux

We employed two platforms for the cohort database system, as illustrated in Fig. 2. For longitudinal data collection and management, we selected a stable, reliable, and confidential database platform. Utilizing the internet based clinical research and trial management system (iCReaT, version 2) by the Korea Disease Control and Prevention Agency, we collaborated with medical staff to collect clinical data from EMRs. Simultaneously, Moaform, an online survey platform, facilitated the collection of demographics, health self-management, and psychosocial well-being data through patient-reported outcome measures (PROMs) from cohort children and parents.

#### Cohort quality control

Established before recruitment, the cohort quality control plan was overseen by the principal investigator. Two researchers validated weekly cohort quality and reported to the principal investigator. Questionnaires, derived from the theoretical framework and literature review, were developed in consultation with the multidisciplinary team at the SB clinic. To ensure the completeness of the cohort data, we implemented logic allowing sub-questions to vary based on survey responses and required response logic to prevent non-response. A guide for recruitment and data collection was developed, and research assistants were trained accordingly. We used iCReaT, the electronic case report form (eCRF) platform of the Korea Disease Control and Prevention Agency, to manage EMR data. The principal investigator and three research assistants received training in data entry, correction, and e-CRF validation. Response rates were tracked weekly during data collection. Mobile phone gift cards incentivized survey completion, and non-respondents were encouraged to participate. After data collection, we validated raw data accuracy and completeness, cleaning, and coding according to a pre-established codebook. Statistical indicators for the first year's results were analyzed and plans for the second-year recruitment and data collection were made.

### Initial implementation

#### Study site

This prospective cohort study was conducted at the SB clinic of Severance Children's Hospital, a tertiary care hospital with the largest SB clinics in Seoul, South Korea.

#### Study participants

The cohort included all children aged 4–12 years with a diagnosis of SB and their parents. Inclusion criteria required mutual understanding of the study's purpose and methods, with informed consent. We divided the cohort into preschool age 4–6 years and school age 7–12 years according to developmental stage [25]. Exclusion applied if withdrawal or consistent non-response occurred; data collection ceased upon withdrawal.

#### Sample size

Approximately 600 children aged 4–12 years were enrolled at the SB clinic at Severance Children's Hospital. We targeted 300 eligible children, 50% of the total, with 300 parents, reaching a total of 600. A 50% recruitment rate was deemed feasible, considering the children’s visit of only 1–2 years at the SB clinic.

#### Recruitment

A recruitment notice describing eligibility, purpose, methodology, and compensation for participation was posted on the outpatient bulletin board at Severance Children's Hospital. Participants were provided with a mobile gift card worth 30,000 KRW (approximately 22 USD) for completing the annual survey. Pediatric urologists and PNPs explained the study to eligible patients, emphasizing voluntary participation and the right to withdraw. Verbal consent was obtained from children aged 4–6 years, and written consent from both all parents and children aged 7–12 years. Additional recruitment was planned for years 2 and 3 due to children’s less frequent outpatient visits at the SB clinic.

#### Data collection

Two approaches were used to collect data: (1) diagnosis and clinical outcomes (e.g., lesion level, surgical history, laboratory results) were collected from the EMR by PNPs and (2) demographics, psychosocial characteristics and family data were collected from online self-reported questionnaires completed by children with SB and their parents if the child with SB was aged 7–12 years and by only the parents if the child with SB was aged 4–6 years.

#### Outcome variables

Derived from Swanson's (2010) Life Course Model for Spina Bifida (Fig. 1), this model includes inputs, outputs, self-management/health, personal/social relationships, employment/income support, and family factors. Inputs encompass personal characteristics of children with SB, involving demographic and clinical features. Outputs comprise SB-specific HRQoL, generic HRQoL, and health outcomes such as renal function, urinary and fecal incontinence, and pressure ulcers. Three domains vary by developmental stage: preschool, school-age, and adolescence—assessing self-management/health, personal/social relationships, and employment/income support. ‘Self-management/health’ gauges transition readiness across all three stages, ‘personal/social relationships’ evaluates social support at all stages and introduces SB-related sexuality knowledge and worries in adolescence. ‘Employment/income support’ scrutinizes common childcare or school adjustment, incorporates academic self-efficacy for both school age and adolescence, and adds career preparation behaviors for adolescence. Finally, family factors, impacting all developmental stages and domains, include family resilience, parenting stress, parental depression, and parental HRQoL. The details of the measures are described in Additional file 1.

#### Data analysis

Using SPSS version 27.0 (IBM Corp., Armonk, NY, USA), data analyses explored demographic and clinical characteristics of study participants through descriptive statistics, such as frequencies, percentages, means, and standard deviations.

## Results

### Cohort participants

Between September 30, 2022 and September 30, 2023, 162 children aged 4–12 years and their parents were enrolled in the cohort. Response rates were 93.5% (58/62) for children aged 4–6 years and 94.5% (104/110) for those aged 7–12 years. Eleven participants withdrew consent, and six children were considered withdrawn due to consistent non-response. Four children declined after initial consent, and one child withdrew before Year 2, with Year 1 data included in the analysis.

### Clinical characteristics of the cohort

Clinical characteristics included neurosurgical, bladder management and urological, bowel management and general surgical, and mobility and orthopedic characteristics.

### General and neurosurgical characteristics of the cohort

The mean age of the cohort children (*N* = 162) was 7.9 ± 2.6 years, with 35.8% (*n* = 58) aged 4–6, 29.0% (*n* = 47) aged 7–9, and 35.2% (*n* = 57) aged 10–12. Lipomyelomeningocele (LMMC) was the most common diagnosis (51.2%, n = 83), followed by MMC (27.2%, *n* = 44), and tethered cord syndrome (TCS) (20.4%, *n* = 33). Of the four classifications of LMMC (*n* = 83), caudal (33.7%) was the most common, followed by dorsal (27.7%). Almost all children (98.8%) had a history of neurosurgery, with only seven (4.3%) having a ventriculoperitoneal (VP) shunt (Table 1).

**Table 1:** General and neurosurgical characteristics of the cohort (*N* = 162)

Variable	n (%)
Age (year), M (SD)	7.9 (2.6)
4–6	58 (35.8)
7–9	47 (29.0)
10–12	57 (35.2)
Sex	
Girl	92 (56.8)
Boy	70 (43.2)
Spina bifida type	
MMC	44 (27.2)
Non-MMC	118 (72.8)
Type of non-MMC (*n*=118)	
LMMC	83 (51.2)
TCS	33 (20.4)
Other	2 (1.2)
Classification of LMMC (n = 83)	
Dorsal	23 (27.7)
Transitional	13 (15.7)
Chaotic	4 (4.8)
Caudal	28 (33.7)
Unknown	15 (18.1)
Neurosurgical history^a^	160 (98.8)
MMC repair	43 (26.5)
LMMC repair	82 (50.6)
VP shunt	7 (4.3)
Other	49 (30.2)

*LMMC* lipomyelomeningocele, *MMC* myelomeningocele, *TCS* tethered cord syndrome, *VP* ventriculoperitoneal

^a^Multiple response

### Bladder management and urological characteristics of the cohort

CICs were used by 38.3% (*n* = 62) of the cohort, and 46.2% (*n* = 74) took bladder medication. Healthcare providers recommended a mean of 4.8 ± 1.3 CICs per day, with 88.7% (55 out of 62 participants) aware of this. Among CIC users (*n* = 62), self-CICs (*n *= 27) increased with age: 4–6 years (*n* = 2), 7–9 years (*n* = 10), and 10–12 years (*n* = 15), while parent-CICs (*n* = 35) decreased with age. The mean age for children to start self-CIC was 7.6 ± 1.7 years (range: 5–12 years). Parents reported that the main reason for performing CIC was because the child was young. In addition, 50 participants (30.9%) had urinary incontinence with an average of 3.6 ± 2.9 episodes/day. Four children (2.5%) had latex allergy and 70 children (43.2%) did not know whether they had latex allergy. Urological complications included UTI in 16%, VUR in 13%, and hydronephrosis in 11.7%. In total, 37 children (22.8%) had a history of urological surgery, mainly vesicoureteral anastomosis (8.6%) and vesicoureteral reflux repair (6.2%) (Table 2).

**Table 2:** Bladder management and urological characteristics of the cohort (*N* = 162)

Variable	n (%)	Range
Bladder management^a^		
Spontaneous void	113 (69.8)	
CIC	62 (38.3)	
Bladder medication (antimuscarinic, antibiotics etc.)		
Yes	74 (45.7)	
No	88 (54.3)	
Awareness of recommended daily CIC frequency (*n* = 62)		
Yes	55 (88.7)	
No	7 (11.3)	
Recommended number of CIC (*n* = 55), M (SD)	4.8 (1.3)	2–7
Performer of CIC (*n* = 62)		
Parent	35 (56.5)	
4–6 years	21 (33.9)	
7–9 years	10 (16.1)	
10–12 years	4 (6.5)	
Child	27 (43.5)	
4–6 years	2 (3.2)	
7–9 years	10 (16.1)	
10–12 years	15 (24.2)	
Age when child started practice CIC (*n* = 27), M (SD)	7.6 (1.7)	5–12
Urinary incontinence		
Yes	50 (30.9)	
No	112 (69.1)	
Number of urinary incontinence per day (*n* = 50), M (SD)	3.6 (2.9)	0.14–12.0
Latex allergy		
Yes	4 (2.5)	
No	88 (54.3)	
Unknown	70 (43.2)	
Urological complications^a^		
Hydronephrosis	19 (11.7)	
UTI	26 (16.0)	
VUR	21 (13.0)	
Urological surgical history^a^	37 (22.8)	
Bladder augmentation	1 (0.6)	
Appendicovesicostomy/Mitrofanoff	2 (1.2)	
Vesicostomy closure	4 (2.5)	
Vesicoureteral reflux repair	10 (6.2)	
Vesicoureteral anastomosis	14 (8.6)	
Orchiopexy	2 (1.2)	
Vesicostomy	6 (3.7)	
Intravesical Botox injection	2 (1.2)	
Other	14 (8.7)	

*CIC* clean intermittent catheterization, *UTI* urinary tract infection, *VUR* vesicoureteral reflux

^a^Multiple response

### Bowel management and general surgical characteristics of the cohort

Regarding bowel management and general surgical characteristics (Table 3), 36 (22.2%) of children used enemas and 25 (15.4%) used laxatives. The mean daily defecations were 1.3 ± 1.7, with enemas performed 3.3 ± 2.6 times weekly. Among enema users (*n* = 36), cone enemas were most common (63.9%), followed by glycerin enemas (25%). Parents conducted most enemas (88.9%), often due to their young age, while only four children (11.1%) aged 10–12 years performed enemas themselves. Children began self-administering enemas at a mean age of 8.8 ± 3.0 years. Additionally, 26.5% (*n* = 43) of children with SB had fecal incontinence. The mean weekly episodes were 1.9 ± 2.6, with 3.1 ± 6.1 pads or diapers used weekly. Surgery was performed in 20.4% of patients, predominantly colostomy (11.1%) and posterior sagittal anorectoplasty (6.8%).

**Table 3:** Bowel management and general surgical characteristics of the cohort (*N* = 162)

Variable	n (%)	Range
Bowel management^a^		
Spontaneous defecation	132 (81.5)	
Enema	36 (22.2)	
Laxatives	25 (15.4)	
Number of defecations per day, M (SD)	1.3 (1.7)	0–13
Number of taking laxatives per week (*n* = 25), M (SD)	3.6 (2.4)	0.5–7.0
Number of performing enema per week (*n* = 36), M (SD)	3.3 (2.6)	0.13–14.0
Type of enema (*n* = 36)^a^		
Cone enema	23 (63.9)	
Glycerin enema	9 (25.0)	
Antegrade continence enema	4 (11.1)	
Anus massage	2 (5.5)	
Peristeen enema	0 (0.0)	
Performer of enema (*n* = 36)		
Parent	32 (88.9)	
4–6 years	11 (30.5)	
7–9 years	10 (27.9)	
10–12 years	11 (30.5)	
Child	4 (11.1)	
4–6 years	0 (0.0)	
7–9 years	0 (0.0)	
10–12 years	4 (11.1)	
Age when child started practice enema (*n* = 4), M (SD)	8.8 (3.0)	5–12
Fecal incontinence		
Yes	43 (26.5)	
No	119 (73.5)	
Number of fecal incontinence episodes per week (*n* = 43), M (SD)	1.9 (2.6)	0.1–15
Number of pads/diapers used for fecal incontinence per week (*n* = 43), M (SD)	3.1 (6.1)	0–30
General surgical history^a^	33 (20.4)	
Malone antegrade continence enema (MACE)	2 (1.2)	
One stage anoplasty	3 (1.9)	
Colostomy formation	18 (11.1)	
Colostomy repair	2 (1.2)	
Posterior sagittal anorectoplasty (PSARP)	11 (6.8)	
Soave operation	1 (0.6)	
Duhamel operation	0 (0.0)	
Other	20 (12.3)	

^a^Multiple response

### Mobility and orthopedic characteristics of the cohort

Regarding mobility and orthopedic characteristics (Table 4), 144 (88.9%) children with SB were able to walk independently without assistance, and 24 (14.8%) ambulated with an assistive device, such as an orthosis, walker, or wheelchair. Concerning orthopedic deformities, 16 (9.9%) children had a spinal deformity, 5 (3.1%) had a hip deformity, 7 (4.3%) had a knee deformity, 6 (3.7%) had a leg length discrepancy, and 39 (24.1%) had foot deformities. Foot deformities (*n* = 39) were most common in both feet (46.2%), with equinus being the most common type (46.2%). Only one child experienced a pressure sore on the hip and leg twice in the past year. Additionally, 6.8% of the children underwent orthopedic surgery, with 4.3% below the knee.

**Table 4:** Mobility and orthopedic characteristics of the cohort (*N* = 162)

Variable	n (%)
Mobility^a^	
Independently ambulation	144 (88.9)
Wheelchair or walker	5 (3.1)
Orthosis	19 (11.7)
Type of orthosis (*n* = 19)	
Ankle Foot Orthoses (AFO)	14 (73.7)
Knee-Ankle–Foot Orthosis (KAFO)	5 (26.3)
Orthopedic deformity^a^	
Spinal deformity	16 (9.9)
Hip deformity	5 (3.1)
Knee deformity	7 (4.3)
Leg length discrepancy	6 (3.7)
Foot deformity	39 (24.1)
Side of foot deformity (*n* = 39)	
Both feet	18 (46.2)
Right foot	16 (41.0)
Left foot	5 (12.8)
Type of foot deformity (*n* = 39)^a^	
Equinus	18 (46.2)
Calcaneus	13 (33.3)
Varus	12 (30.8)
Valgus	4 (10.3)
Orthopedic surgical history^a^	11 (6.8)
Above the knee	1 (0.6)
Below the knee	7 (4.3)
Other	5 (3.1)

^a^Multiple response

## Discussion

### Principal results

In this single-center prospective cohort study, we have shown feasibility by establishing and initially implementing a cohort of children with SB based on the Life Course Model for Spina Bifida. Despite recruiting about half of the planned participants (162/300, 54%), we achieved a high response rate (94%). Considering the regular check-ups for children with SB every 1–2 years, our recruitment efforts will continue. With parents also involved, we included 162 families in the Year 1 cohort.

Analysis of baseline data from the Year 1 cohort allowed us to identify typical clinical characteristics of Korean children with SB aged 4–12 years. Regarding SB type, non-MMC was more common than MMC, with less than 5% having VP shunt in situ or using a wheelchair, contrasting the clinical distribution in the NSBPR in the United States [15]. However, the proportion of children using CIC and enema for bladder and bowel management, urinary incontinence, and fecal incontinence was comparable to the NSBPR [15].

### Comparison with prior work

We developed the cohort using the Life Course Model of Spina Bifida as the theoretical framework, selecting appropriate variables for its subdomains: inputs, self-management/health, personal/social relationships, employment/income support (major life areas), family factors, and outputs [9].

Inputs included SB type, bladder and bowel management, history of surgery, laboratory findings, and complications based on NSBPR’s initial encounter forms and annual visit forms [11]. Outcome measures from previous SB studies [3, 4, 15, 26, 28–30] were also included.

In the self-management/health domain, we assessed transition readiness using the Transition Readiness Assessment Questionnaire-SB (TRAQ-SB) measuring daily activities for transition such as tracking health issues and communicating with healthcare providers, as well as SB-specific self-management, such as bladder and bowel management [31]. Transition readiness is important as a mediator of successful transition to adult health care, and self-management is a predictor of transition [7]. In terms of personal/social relationships, we assessed social support from family, friends, and teachers [32]. Social support has been known to promote independence in children with SB [33]. Additional questions in adolescence covered romantic relationships, puberty characteristics, SB sexuality knowledge, and SB worries scale [34]. Sexuality is a developmentally appropriate variable that should be addressed before and during puberty [35]. In terms of employment/income support, we commonly assessed childcare/school adjustment [36] and added academic self-efficacy in both school age and adolescence [37], and career preparation behaviors in adolescence [38]. Major life areas are often overlooked in SB care, yet vital as the focus shifts to HRQoL and participation [9, 38–41].

Outputs included renal function, urinary/fecal incontinence, pressure ulcers that are known as major health outcomes in people with SB [26], and HRQoL the ultimate goal of care for people with SB [5]. HRQoL was assessed using the Korean versions of QUALAS-C and QUALAS-T [22, 23], which are age-appropriate and SB-specific [42, 43], in conjunction with the Korean version of KIDSCREEN-27 [44], a reliable and valid instrument for assessing HRQoL in children with more extensive chronic conditions [45], to allow comparison with other children with chronic conditions.

Family factors included family resilience, parenting stress, parental depression, and HRQoL [46–49]. Family resilience was a protective factor, while parenting stress and parental depression were detrimental to HRQoL in children with chronic conditions [50, 51]. Parental factors play a crucial role in facilitating or hindering child's responsibility for self-management, independence, and transition to adulthood [8, 19].

The clinical distribution of Korean children in this cohort, including the proportions of SB type, VP shunt in situ, use of CIC/enema, urinary/fecal incontinence, and ambulatory status, was consistent with that of previous studies on children with SB in Korea; the ratio of MMC to non-MMC (2:8, 3:7, or 4:6), 5 − 10% VP shunt in situ, 40 − 50% CIC, 20 − 40% enema, 30 − 40% urinary incontinence, 20 − 40% fecal incontinence, and < 1% pressure sore [18, 22–24, 52]. Compared to the clinical distribution of NSBPR in the United States, the proportions of clinical features are reversed from those in Korea; the ratio of MMC to non-MMC (8:2, 7:3, or 6:4), 60 − 70% VP shunt in situ, 60 − 70% CIC, 30 − 40% enema, 60 − 70% urinary incontinence, 30 − 50% fecal incontinence, 50% community ambulator, 10 − 15% pressure sore [11, 15, 26]. However, it differed from the clinical distribution of NSBPR in the United States due to sociocultural and healthcare system reasons, including the reimbursement of intensive prenatal screening by the national health insurance system in Korea, the possibility of early detection of fetal anomalies due to frequent prenatal testing, high rate of induced abortions for fetuses with abnormalities, free provision of maternal folic acid supplements by community health centers, and discriminatory sociocultural perceptions of disability [53–57]. Therefore, this newly established cohort has demonstrated a typical clinical distribution of children with SB in Korea.

### Research implications

To the best of our knowledge, this is the first cohort study of children with congenital anomalies in Korea. Due to the relative obscurity of congenital anomalies and rare, incurable diseases like SB, scant information exists regarding their precise epidemiology, progression into adulthood, and long-term adult health outcomes in Korea. Existing studies have predominantly focused on chronic diseases in adults and developmental cohorts of healthy children. While previous cohort studies relied on physician-led, EMR-based clinical data, this study showcases the viability of an interdisciplinary medical-nursing cohort that integrates EMR data with self-reported data from children and their families. This cohort can longitudinally and comprehensively discern factors impacting HRQoL in children with congenital anomalies, laying the groundwork for future multidisciplinary cohort studies.

### Limitations

This prospective cohort study was conducted at a single institution, which limits the generalizability of the findings; however, it has the potential for future expansion to a multicenter cohort in collaboration with other SB clinics in Korea.

We aimed to enroll 300 children and their parents, 50% of all children registered at the SB clinic, but only recruited 162 (54%) children and their parents were recruited. We considered 50% to be feasible, because children with SB have regular hospital visits every 1–2 years, depending on their conditions, but this was not realistically possible. We were unable to enroll as many children and their parents as planned for several reasons, including patients skipping regular visits for personal reasons and parents declining study participation due to reluctance to share their child's health information.

To improve cohort recruitment, we have changed our plan and will continue recruitment in years 2 and 3 to ensure that we have a full cohort with no missing participants. We will also ensure that we fully explain privacy during recruitment to allay parents' concerns about providing health information. To maintain the cohort, we created and sent an e-card newsletter to participants at the end of Year 1 cohort recruitment. The e-newsletter included a thank you note, a reminder of the start of Year 2 of the study, and the purpose, methods, Year 1 recruitment results, and significance of this cohort study. We also attempted to retain the cohort by providing them with a mobile phone gift voucher within a week of completing the survey and by contacting them individually to thank them.

## Conclusions

The cohort was developed in collaboration with a multidisciplinary team to monitor the health outcomes of patients attending the largest SB clinic in South Korea. The successful recruitment and data collection underscore the feasibility of cohort studies involving chronically ill children with congenital anomalies. The cohort's purpose is to delineate demographics, clinical characteristics, and psychosocial outcomes of SB-affected children in Korea. It aims to furnish a database platform for epidemiologic and clinical studies and offer evidence for comparing health outcomes to inform future improvements in quality of care. Ongoing data collection for a longitudinal database will validate the natural history of SB, spotlight long-term health outcomes in adults with SB, and serve as a foundation for life-course interventions.

## Supplementary Information

Supplementary Material 1.

## Data Availability

The datasets used and/or analyzed during the current study are available from the corresponding author on reasonable request.

## Abbreviations

CIC: Clean intermittent catheterization
eCRF: Electronic case report form
EMR: Electronic medical record
HRQoL: Health-related quality of life
iCReaT: Internet based clinical research and trial management system
LMMC: Lipomyelomeningocele
MMC: Myelomeningocele
NS: Neurosurgery
NSBPR: National spina bifida patient registry
OS: Orthopedics
PNP: Pediatric nurse practitioner
PROM: Patient-reported outcome measure
SB: Spina bifida
TCS: Tethered cord syndrome
URO: Urology
UTI: Urinary tract infection
VP: Ventriculoperitoneal
VUR: Vesicoureteral reflux
