# Supplementary material for: Clinical profile of Korean children with spina bifida: a single-center prospective cohort study
Source: BMC Pediatr. 2024 Dec 3;24:791. doi: 10.1186/s12887-024-05229-5 (PMC11613806; doi:10.1186/s12887-024-05229-5)
Supplement: Supplementary file 1 — Supplementary Material 1. [file 12887_2024_5229_MOESM1_ESM.docx]

**Additional file 1.** Variables depending on the theoretical framework and developmental stage.

| Domain | Pre-school  (4–6 years) | School-age  (7–12 years) | Adolescence  (13–16 years) |
| --- | --- | --- | --- |
| Inputs | Demographics  Clinical characteristics | Demographics  Clinical characteristics | Demographics  Clinical characteristics |
| Self-management/  health | Transition readiness [31] | **Transition readiness [31]** | **Transition readiness [31]** |
| Personal/social relationships | Social support [32] | **Social support [32]** | **Social support [32]**  **SB sexuality knowledge and worries^†^ [34]** |
| Employment/  income support | Childcare adjustment [36] | **School adjustment [36]**  **Academic self-efficacy^†^ [37]** | **School adjustment [36]**  **Academic self-efficacy^†^ [37]**  **Career preparation behaviors^†^ [38]** |
| Outputs | HRQoL (General) [45]  HRQoL (SB specific) [22] | **HRQoL (General) [45]**  **HRQoL (SB specific) [22]** | **HRQoL (General) [45]**  **HRQoL (SB specific) [23]** |
| Family factors | Family resilience [50]  Parenting stress [46]  Parent’s depression [47]  Parent’s HRQoL [48] | Family resilience [50]  Parenting stress [46]  Parent’s depression [47]  Parent’s HRQoL [48] | Family resilience [50]  Parenting stress [46]  Parent’s depression [47]  Parent’s HRQoL [48] |

*Note.* HRQoL, health-related quality of life; SB, spina bifida; bold font=child-reported variable; regular font=parent-reported variable; ^†^Newly added variable depending on developmental stage.
